# Assessment of utilization of long acting reversible contraceptive and associated factors among women of reproductive age in Harar City, Ethiopia

**DOI:** 10.11604/pamj.2017.28.222.13475

**Published:** 2017-11-10

**Authors:** Kasiye Shiferaw, Abdulbasit Musa

**Affiliations:** 1 Department of Midwifery, College of Health and Medical Sciences, Haramaya University, Ethiopia

**Keywords:** Utilization, long acting reversible contraception, associated factors

## Abstract

**Introduction:**

World health organization report indicated that, in 2013 alone, over 289,000 maternal death that resulted from pregnancy and delivery related complication were reported worldwide indicating a decline of 45% from 1990. The sub-Saharan Africa region alone accounted for 62% of maternal death followed by southern Asian country (24%). Provision of family planning is one of the effective intervention that prevent unwanted and ill spaced pregnancy there by reducing maternal mortality and morbidity. Given that its effectiveness and, associated fewer visits to health facilities, LARC are very important in tackling maternal mortality and morbidity. However, little is known regarding its prevalence in eastern Ethiopia. Thus, this study aimed to assess utilization of long acting reversible contraceptives and associated factors among women of reproductive age groups.

**Methods:**

A facility based cross-sectional study conducted in Harar city among 402 study participants. The study participants selected by using systematic random sampling method. The quantitative data collected using structured interviewer administered questionnaires. All variables with p-value of ≤ 0.25 in bivariate logistic regression were taken into multivariable model. Variables having p value ≤ 0.05 in the multivariate analysis were taken as significant predictors. Crude and adjusted odds ratios with their 95% confidence intervals were calculated.

**Results:**

The study identified that the utilization of long acting reversible contraceptive among mother of reproductive age was 38%. Study participants whose occupation was daily laborer were less likely to utilize long acting reversible contraceptive compared to those whose occupation was house wife (adjusted OR = 0.3; 95% CI 0.01 to 0.8). Moreover, those mothers who were unable to read and write utilize long acting reversible contraceptive 5 times more likely compared to those who were above grade 12 (adjusted OR = 4.9; 95% CI 1.2 to 19.6).

**Conclusion:**

The prevalence of long acting reversible contraceptive was found to be low. Maternal education and occupation were factors found to have a significant association with utilization of long acting reversible contraceptive. Community and facility level awareness creation should be reinforced to improve utilization of long acting reversible contraceptives.

## Introduction

Globally, there were an estimated 289,000 maternal deaths in 2013, a decline of 45% from 1990. The sub-Saharan Africa region alone accounted for 62% (179,000) of global deaths followed by Southern Asia at 24% (69 000). The global MMR in 2013 was 210 maternal deaths per 100,000 live births, down from 380 maternal deaths per 100 000 live births in 1990. The MMR in developing regions (230) was 14 times higher than in developed regions [1]. Fortunately, the vast majority of maternal and newborn deaths can be prevented with proven interventions to ensure that every pregnancy is wanted using modern contraceptive and every birth is safe [2]. The unmet need for FP in Ethiopia decreased from 34% in 2005 to 25% in 2011. A disparity exists between contraceptive use rates in rural and urban areas, with less than half as many women in rural areas as in urban areas using modern methods (22% vs nearly 50%). The Total Fertility Rate in Ethiopia declined from 6.4 children per woman in 1990 to 5.4 in 2005 and 4.8 in 2011. Despite these positive trends, the gap between demand for and uptake of FP is large. While nearly 75% of currently married women of reproductive age report a desire to delay childbirth for at least two years or stop bearing children altogether, only 27.3% of them are currently using a modern contraceptive method to prevent pregnancy. Use of FP in Ethiopia has traditionally been limited to short-acting methods such as injectables and pills due to limited access to Long-Acting FP (LAFP) methods, commodity shortages and lack of skilled health care providers to offer services at the community level [3]. An estimated 80 million unintended pregnancies occur each year worldwide, resulting in 42 million induced abortions and 34 million unintended births. These unintended pregnancies have grave consequences for the health and well-being of women and families, particularly in low and middle-income countries where maternal mortality is high and abortions are often unsafe. Three hundred and fifty-eight thousand women die of pregnancy-related causes every year, many resulting from unintended pregnancies that were unsafely aborted [4]. The rate of unintended pregnancy is high across the world. Unintended pregnancy is not only resulted in substantial costs to health services, it can lead to personal distress for women experiencing it. Whilst a large number of unintended pregnancies occur in those women who are not using any method of contraceptive, it can also occur in women using a contraceptive method incorrectly or inconsistently [5].

Unintended pregnancy remains a significant global public health problem; its rate is greater in less developed regions (57 per 1000 women aged 15-44 years) than in more developed regions (42 per 1000). In real-world tests LARC methods were over 20 times more effective at preventing unintended pregnancy compared to the contraceptive pill, patch or ring. Although LARC use and continuation has been proven to effectively reduce unintended pregnancy thereby reducing abortion, not more than 15% of women use LARC method worldwide [6]. The most effective methods of contraceptive are frequently the least available [7]. In spite of their effectiveness, LARC are under-utilized by women. Educating women and health professionals and dispelling myths about these methods may improve their acceptability. Furthermore, facilitating uptake by ensuring that a range of contraceptive providers are trained and able to provide to women without undue delay may also be effective strategies to improve uptake and prevent more unintended pregnancies [8]. Repeat pregnancy within 2 years of a previous birth or abortion occurs in approximately 35% of recently pregnant female adolescents. Rapid repeat pregnancy (RRP) is associated with increased maternal and neonatal morbidity and continues a cycle of economic deprivation for young women and their families. Adolescents who do not initiate a LARC method have up to a 35 times increased risk of RRP compared with their peers using LARC [9]. A decrease in unmet need for family planning accompanied this rapid increase in the use of contraceptives, such that 12 per cent of married or in-union women globally had an unmet need for family planning in 2015. However, wide disparities in the level of unmet need for family planning are still evident among countries, and a benchmark set by Governments in 1999 to close the gap in meeting demand for family planning by 2015 is out of reach for most countries [10].

Overall modern contraceptive prevalence has increased in Sub-Saharan Africa (SSA). The ongoing increase in the contraceptive use is due to changes in behavior consistent with the ongoing family planning promotion over the past 30 years. By contrast, an increase in the proportion of women with secondary education does not explain the change in modern contraceptive prevalence in most SSA countries [11]. Ethiopia is one of the Sub-Saharan African countries with highest MMR which is 676 maternal deaths per 100,000 live births. The vast majority of maternal and new born deaths can be prevented with proven interventions to ensure that every pregnancy is wanted by using the most effective modern Family planning methods and every birth is safe [3]. Holistic, client-centered approaches to FP programming create effective and successful programs that ultimately support health systems to meet the RH intentions of women and men. Although LARC decreases unintended pregnancies by eliminating user compliance issues, its uptake is low. Strategies that promote LARC uptake by targeting specific barriers may effectively reduce high unintended pregnancy rate [12]. Long acting reversible methods of contraceptives are the most effective methods of contraceptives. In spite of this, they are under-utilized by women in developed countries. Educating women and health professionals and dispelling myths about these methods may improve their acceptability. Furthermore, facilitating uptake by ensuring that a range of contraceptive providers are trained and able to provide to women without undue delay, particularly in the immediate post abortion and postpartum period, may also be effective strategies to improve uptake and prevent more unintended pregnancies [5]. For fecund, sexually active women, LARC offers an effective way to postpone childbearing for relatively long periods of time. Women in Ethiopia could benefit significantly from greater knowledge and availability of these methods and family planning programs and policies targeted at serving women should emphasize these methods more strongly [13]. Long acting reversible contraceptive is least used family planning method in Ethiopia. There was no research conducted in the study area to assess utilization and factors associated with use of long acting reversible contraceptive among women of reproductive age. Therefore, this study aimed to assess utilization of long acting reversible contraceptives and associated factors among women of reproductive age groups in Harar city. The finding of the study is an input for policy makers to make intervention that help in reduction of maternal mortality and morbidity through speeding up utilization of LARC method.

## Methods

A facility based cross-sectional study was conducted from November, 2015 to January, 2016. The study was done in Harar city. The town is 524km away from Addis Ababa at an elevation of 1,885 meters. Based on figures from the Central Statistical Agency in 2007, Harar has an estimated total population of 122,000, of whom 60,000 were male and 62,000 were females (CSA National Statistics 2007). The Harar city has two public Hospitals (Jugal hospital and Hiwot Fana specialized university hospital), Police Hospital, Army Hospital, two private hospitals (Harar General Hospital and Yemaje Hospital), eight health centers and one regional public health laboratory. The health service coverage of the region reached to 100% according to the federal ministry of health (Harari Regional Health Bureau, 2010). A single population proportion formula was used assuming expected prevalence of long acting reversible contraceptive use of 19.5% which is taken from previous study [14] and marginal error (0.04) and by adding 10% non-response, the final sample size was 402. Both public and private health facilities are providing family planning services in the city. For this study only government health facilities was considered, i.e. one hospital and two health center of these government health facilities were included in the study. The calculated sample size was proportionally allocated to each health facility based on the previous consecutive three months average daily client flow of the units which was obtained by referring client registration log books. The average monthly client flow of women in all selected health facilities was found to be 120 in a month. Health institutions were selected using simple random sampling technique. The study participants were selected by using systematic random sampling method from family planning service users who visited the health facilities for the use of contraceptive during the data collection period. The first client in each health facility was selected by lottery method.The source population was all women of reproductive age group visiting health facilities in Harar city for family planning service. All women of reproductive age group (15-49 years) who were taking family planning at selected health institutions were included in the study. Women who were sick and cannot respond to the interview questionnaire were excluded.

The dependent variable of the study was utilization of long acting reversible contraceptive and independent variable were socio-demographic variables (age, religion, ethnicity, residence, educational status, husband educational status, income, woman occupation, husband occupation), obstetric factors (parity, age at first marriage, age at first birth, history of abortion, complication related to pregnancy, number of children woman wish to have), awareness variables (discussed contraceptive, heard about long term reversible contraceptives). The quantitative data was collected using structured interviewer administered questionnaires. Questionnaires constituted information on socio-demographic, obstetric factors variables, and practice of LARCs questions. The questionnaire was adapted from different studies considering the local situation of the study area. The questionnaire was first prepared in English then translated to local language Afan Oromo for data collection by language expert. To check whether the translation was consistent with the English version the questionnaire was back translated to English by another language expert. Before the actual data collection, the questionnaire was pre-tested on 5% of family planning user from unselected health facility. Based on the pretest, the time needed for completing interview, and the number of data collectors were estimated. Moreover; the questionnaire was corrected accordingly. The principal investigators trained five diploma midwives as data collectors and bachelor midwives as supervisors for two consecutive days on the objective of the study, data collection tools and interview techniques. The interview conducted in a place where the woman feels free to express her feelings and ideas. The questionnaires checked by the supervisors on daily basis for completeness. The quantitative data was entered, cleaned using Epi info version 3.5.4 and transferred to Statistical package for social sciences (SPSS) version 20.0 for analysis. First descriptive analyses was carried out for each of the variables; Second, bivariate analyses was done for the independent variables with the outcome variable to select candidate variables for the multivariable analyses. Finally, all variables with p-value of ≤ 0.25 in bivariate logistic regression were taken into multivariable model. Variables having p value ≤ 0.05 in the multivariate analysis were taken as significant predictors. Crude and adjusted odd ratios with their 95% confidence intervals were calculated. Ethical clearance obtained from St. Paul millennium teaching hospital and written formal letter was given to respective institutions where the study was conducted.

## Results

**Socio-demographic characteristics:** There were 400 participants included in the study and response rate for the study was over 99%. The mean age of the women was 26.7 (SD = 5.8) years. About 47.8 % respondents were Muslim and majorities of study participants (62.8%) are Oromo ethnic group. More than fifty five percent of respondents were from urban, about 75.8% had attended formal education and (43.5%) were house wives. About 84.4% of the respondents' husband was attended formal education and 38.5% were government employed (Table 1).

**Table 1:** Socio-demographic and obstetrics characteristics of women of reproductive age in Harar city, 2016

Variable		Frequency	Percent
Age	< 18	25	6.3
	18 - 24	121	30.3
	25 – 34	206	51.5
	≥ 35	48	12
Religion	Orthodox	129	32.3
	Muslim	191	47.8
	Protestant	74	18.5
	Other(specify)	6	1.6
Ethnicity	Oromo	251	62.8
	Amara	74	18.5
	Harari	46	11.5
	Tigrae	19	4.8
	Others	10	2.5
Residence	Urban	222	55.5
	Rural	178	44.5
Woman educational status	Unable to read & write	46	11.5
	Able to read and write only	49	12.3
	Grade 1-6	100	25
	Grade 7-12	59	14.8
	Above grade 12	52	13
Husband educational status	Unable to read & write	21	5.25
	Able to read and write only	42	10.5
	Grade 1-6	53	13
	Grade 7-12	135	33.75
	Above grade 12	149	37.25
Women occupation	House wife	174	43.5
	Employed (private)	28	7
	Employed (government)	59	14.75
	Small scale merchant	52	13
	Farmer	22	5.5
	Daily laborer	28	7
	Student	37	9.25
Husband occupation	Employed (private)	38	9.5
	Employed ( gov’t)	154	38.5
	Small scale merchant	65	16.25
	Farmer	90	22.5
	Others	53	13.25
Age at first marriage	< 18	108	27
	≥ 18	292	73

**Figure 1:**
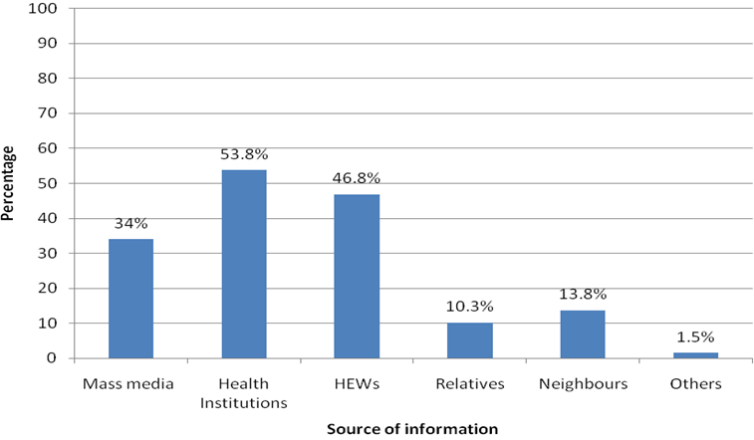
Source of information of long acting reversible contraceptive of reproductive age women in Harar city, 2016

**Figure 2:**
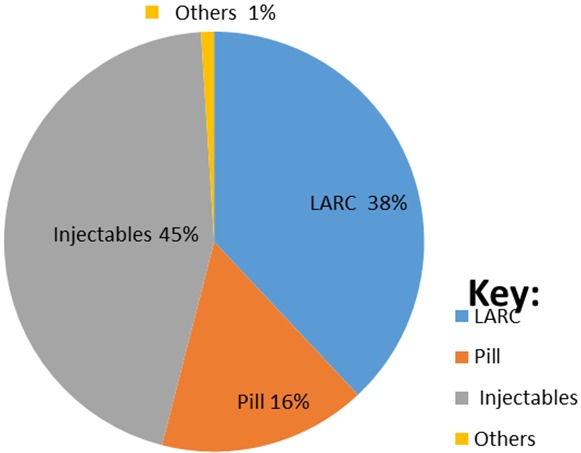
Short and long acting reversible contraceptive used by study participants in Harar city, 2016

**Practice of long acting reversible contraceptive:** Majorities of study participants (86.3%) discussed contraceptives with their husband. More than 89 % of the interviewees have heard about long acting reversible contraceptive and their major source of information was health institutions (Figure 1). More than half of the study participants (52%) have heard about both implants and intrauterine device and only 23% heard about implants. Study has found that utilization of long acting reversible contraceptive was 38% and implants were used by most of study participants (31%). The decision to utilize contraceptive was made by both husband and wife (44%) followed by husband (38%), but only 17.5% of study participants decided by themselves to utilize contraceptive. About 30.5% and 20% study participants didn't use long acting reversible contraceptive because they were using short term contraceptive and fear of side effect respectively. Majority of study participants (76.3%) has intentions to use long acting reversible contraceptive and most of them (57.3%) have the intention to use implants. The study also revealed that about 45% of study participants have utilized inject able contraceptive method (Figure 2).

**Factors associated with utilization long acting reversible contraceptives:** in the bivariate analysis age, religion, ethnicity, educational status of woman and husband, occupation of woman and husband, age at first marriage and discussing contraceptive were found to be significantly associated with utilization of long acting reversible contraceptive (P-value < 0.2). The result of multiple logistic regression models revealed that religion, ethnicity, educational status of woman and occupation of woman were variables significantly associated with utilization of long acting reversible contraceptive. Study participants whose occupation was daily laborer were less likely to utilize long acting reversible contraceptive compared to those whose occupation was house wife (adjusted OR = 0.3; 95% CI 0.01 to 0.8). Moreover, those mothers who were unable to read and write utilize long acting reversible contraceptive 5 times more likely compared to those who were above grade 12 (adjusted OR = 4.9; 95% CI 1.2 to 19.6) (Table 2).

**Table 2:** Bivariate and multivariate analysis of characteristics of utilization of LARC among reproductive age women in Harar city, 2016

Variable		Use LARC		Crude OR(CI)		P-value	Adjusted OR(CI)		P-value
		Yes	No						
Age	< 18	6	19	1			1		
	18 - 24	44	77	0.5	(0.2, 1.5)	0.2	0.6	(0.2, 2.0)	0.46
	25 - 34	85	121	0.5	(0.2, 1.2)	0.1	0.5	(0.1, 1.8)	0.36
	≥ 35	17	31	0.6	(0.2, 1.7)	0.3	0.7	(0.1, 3.0)	0.65
Religion	Orthodox	48	81	1			1		
	Muslim	58	133	1.4	(0.8, 2.2)	0.2	0.8	(0.4, 1.6)	0.73
	Protestant	42	32	**0.5**	**(0.2, 0.8)**	**0.007**	**0.4**	**(0.2, 0.9)**	**0.03**
	Catholic	2	1	0.3	(0.02, 3.4)	0.32	0.2	(0.01, 2.7)	0.23
	Other	2	1	0.3	(0.02, 3.4)	0.32	0.1	(0.01, 2.4)	0.18
Ethnicity	Oromo	105	146	1			1		
	Amhara	26	48	1.3	(0.7, 2.2)	0.3	1.7	(0.9, 3.4)	0.09
	Others	21	54	**1.8**	**(1.0, 3.2)**	**0.03**	**2.6**	**(1.2, 5.5)**	**0.01**
Residence	Urban	85	137	0.9	(0.6, 1.4)	0.8	0.6	(0.4, 1.1)	0.16
	Rural	67	111	1			1		
Woman educational status	Unable to read & write	7	39	5	(1.9, 12.6)	0	4.9	(1.2, 9.6)	0.02
	Able to read and write only	12	37	2.7	(1.2, 6.1)	0.01	1.9	(0.5, 6.5)	0.27
	Grade 1-6	34	66	1.7	(0.9, 3.2)	0.07	1.3	(0.5, 3.2)	0.52
	Grade 7-12	64	67	0.9	(0.5, 1.6)	0.83	0.6	(0.3, 1.3)	0.23
	Above grade 12	35	39	1			1		
Husband educational status	Unable to read & write	5	16	2.6	(0.9, 7.5)	0.07	0.4	(0.09, 2.0)	0.3
	Able to read and write only	9	33	**2.9**	**(1.3, 6.6)**	**0.008**	0.7	(0.2, 2.3)	0.58
	Grade 1-6	18	35	1.5	(0.8, 3.0)	0.16	0.5	(0.2, 1.4)	0.24
	Grade 7-12	53	82	1.2	(0.7, 2.0)	0.33	0.6	(0.3, 1.3)	0.27
	Above grade 12	67	82	1			1		
Occupation of husband	Employed (private)	16	22	1					
	Employed (gov’t)	67	87	0.7	(0.3, 1.8)	0.54	1	(0.4, 2.3)	0.92
	Small scale merchant	24	41	0.7	(0.3, 1.3)	0.33	1.3	(0.4, 3.6)	0.59
	Farmer	26	64	0.9	(0.4, 2.0)	0.9	1	(0.3, 3.1)	0.88
	Others	19	34	1.3	(0.6, 2.8)	0.38	1.4	(0.5, 4.1)	0.43
Occupation of woman	House wife	55	119	1					
	Employed (private)	14	14	0.4	(0.2, 1.0)	0.06	0.5	(0.2, 1.4)	0.22
	Employed (government)	31	28	**0.4**	**(0.2, 0.7)**	**0.004**	0.6	(0.2, 1.4)	0.3
	Small scale merchant	18	34	0.8	(0.4, 1.6)	0.68	0.9	(0.4, 1.9)	0.8
	Farmer	4	18	2	(0.6, 6.4)	0.2	1.9	(0.5, 6.9)	0.32
	Daily laborer	17	11	**0.2**	**(0.1, 0.6)**	**0.004**	0.3	(0.1, 0.8)	0.02
	Student	13	24	0.8	(0.4, 1.8)	0.67	0.9	(0.3, 2.2)	0.85
Age at first marriage	**< 18**	31	77	**1.7**	**(1.0, 2.8)**	**0.02**	1.1	(0.6, 2.0)	0.66
	≥ 18	121	171	1			1		

NB: Those written in bold have statistical significant association

## Discussion

The study identified that the utilization of long acting reversible contraceptive among mother of reproductive age was 38%. The study result was higher than study done in Mekele (12.3%), Goba (8.7%), Debremarkos (19.5%), Dendi (17.6%), Arbaminch (13.1%) [ 14-18]. The difference may be due to aggressive community mobilization and awareness creation on long acting contraceptive currently. The comprehensive long acting reversible contraceptive training was given to health care providers and the government is working on accessibility of service. Study participants whose occupation was daily laborer were less likely to utilize long acting reversible contraceptive compared to those whose occupation was house wife. The study has difference with study done in West Ethiopia whose occupation has positive association with LARC [19]. This may be due to daily laborer may be prone to rumors of long acting reversible contraceptive at work place and there are rumors of long acting reversible contraceptive need additional food and not fit for daily laborer. Moreover; those mothers who were unable to read and write utilize long acting reversible contraceptive 5 times more likely compared to those who were above grade twelve. On the other hand study held in West Arsi showed that educational status of being completed grade twelve compared to illiterate were found positively associated with long acting contraceptive methods use with the odds of educational status of women [20]. This may be due to the fact that uneducated women are less resistive of unbalanced approach of some health provider that negotiate clients to some extent to improve utilization of long acting reversible contraceptive coverage in their respective facility.

## Conclusion

The prevalence of long acting reversible contraceptive was found to be low. Maternal education and occupation were factors found to have a significant association with utilization of long acting reversible contraceptive. Community and facility level awareness creation should be reinforced to improve utilization of long acting reversible contraceptives.

### What is known about this topic

- The national prevalence of any modern contraceptive is 27.3% - There is low utilization of long acting reversible contraceptive in Ethiopia - The government of Ethiopia is working on increasing long acting reversible contraceptive utilizations.

### What this study adds

- The prevalence of long acting reversible contraceptive utilization was found to be 38%. Maternal occupation and maternal educational status were factors found to have significant association with utilization of long acting reversible contraception.

## Competing interests

The authors declare no competing interests.

## References

[1] WHO (2016). WHO Maternal health.

[2] USAID (2014). Holistic Approach Enhances Family Planning Programs. Respond's Experience with the SEED Programming Model..

[3] CSA-Ethiopia IC (2012). International: Ethiopia Demographic and Health Survey 2011..

[4] World Health Organization (2010). Trends in maternal mortality: 1990 to 2008: estimates developed by WHO, UNICEF, UNFPA and the World Bank.

[5] Michie L, Cameron S (2013). Improving the uptake of long acting reversible contraceptive. Minerva Ginecol..

[6] Edizioni Minerva Medica (2013). Long-acting reversible contraceptive: a practical solution to reduce unintended pregnancy. Minerva Ginecologica..

[7] WHO (2012). Policy brief From Evidence to Policy: expanding access to family planning strategies to increase use of long-acting and permanent contraceptive.

[8] Holton S, Rowe H, Kirkman M, Jordan L, Mcnamee K, Bayly C (2016). Long-acting reversible contraceptive: findings from the Understanding Fertility Management in Contemporary Australia survey. Eur J Contracept Reprod Heal Care..

[9] Baldwin MK, Edelman AB (2013). The Effect of Long-Acting Reversible Contraception on Rapid Repeat Pregnancy in Adolescents: a Review. J Adolesc Heal..

[10] United Nation (2015). Trends in Contraceptive Use Worldowide.

[11] Emina JBO, Chirwa T, Kandala N (2014). Trend in the use f modern contraception in sub-Saharan Africa: does women's education matter. Contraception..

[12] Mazza D, Bateson D, Frearson M, Goldstone P, Kovacs G, Baber R (2017). Current barriers and potential strategies to increase the use of long-acting reversible contraception (LARC) to reduce the rate of unintended pregnancies in Australia: an expert roundtable discussion. Aust N Z J Obstet Gynaecol..

[13] Vohra NP D (2012). Long-Acting Reversible Contraceptive Use among Ethiopian Youth. Potential Demand and Strategies for Promotion.

[14] Bulto GA, Zewdie TA, Beyen TK (2014). Demand for long acting and permanent contraceptive methods and associated factors among married women of reproductive age group in Debre Markos Town, North West Ethiopia. BMC Womens Health..

[15] Alemayehu M, Belachew T, Tilahun T (2012). Factors associated with utilization of long acting and permanent contraceptive methods among married women of reproductive age in Mekelle town, Tigray region, north Ethiopia. BMC Pregnancy Childbirth..

[16] Takele A, Degu G, Yitayal M (2012). Demand for long acting and permanent methods of contraceptives and factors for non-use among married women of Goba Town, Bale Zone. South..

[17] Sahilemichael A, Temesgen K (2015). Women's Health Care Determinants of Long Acting Reversible Contraceptives Use among Child Bearing Age Women in Dendi District, Western Ethiopia. Journal of Womens Health Care..

[18] Gultie T (2016). Research Article of long acting contraceptives utilization among Predictors reproductive age women in Arba Minch Zuria district. Quality in Primary Care..

[19] Alemu Sufa, Tesfalidet Tekelab DW (2015). Determinants of long acting and permanent contraceptive methods utilization among married women of reproductive age groups in western Ethiopia: a cross-sectional study. The Pan African Medical Journal..

[20] Zone WA (2017). Journal of Women's Health Care Prevalence and Determinant Factors of Long Acting Contraceptive Utilization among Married Women of Reproductive Age in Adaba Town, West Arsi Zone. Journal of Womens Health Care..

